# Reaching Un-Drugable Intracellular Targets with the Long Arm of Antibodies

**DOI:** 10.18632/oncotarget.1039

**Published:** 2013-05-18

**Authors:** David A. Scheinberg, Tao Dao, Cheng Liu

**Affiliations:** Memorial Sloan-Kettering Cancer Center, New York, New York and Weill-Cornell Medical College; New York, New York; Memorial Sloan-Kettering Cancer Center, New York, New York; Eureka Therapeutics, Inc, Emeryville, California

The vast majority of cellular proteins, many of which are the most interesting and important molecules for regulating normal and neoplastic growth, lie within the cell, hidden from monoclonal antibody (mAb) therapeutics by the barriers of the plasma membrane. We recently demonstrated *in vivo* the successful treatment of several human cancers by use of a human “TCR-like” mAb to the intracellular oncogenic protein, WT1, a difficult to drug cancer target (Dao et al, Science Transl. Med. 5:176r33, 2013.)

Mab have emerged in the last 15 years as some of the most important drugs for cancer, inflammatory diseases and infections. New methods to rapidly select mAb from libraries, to re-engineer mAb with new functionalities, and to make mAb fully human to reduce immunogenicity, has greatly expanded their versatility. The biochemical features of mAb (large size, charge and protein structure) are still an important impediment that restricts diffusion and penetration into cells. Therefore, the exquisite specificity of mAb is prevented from addressing some of the only truly specific cancer targets, such as mutated signaling molecules and transcription factors, fusion-protein oncogenes and many other tumor associated antigens.

How does one use an antibody to reach these interesting targets and kill the cell? MAb have reached intra-cellular targets, but usually after the cell has lysed, releasing histones as an example, or tumor-associated vesicular cargo into the extracellular milieu such as in melanosomal granules, or exposing intracellular proteins by permeabilized membranes. Indeed, there is a FDA-approved mAb imaging agent, Prostascint, that reacts with an intracellular epitope, and thus is only exposed upon death of the cell. In this context, one alternative approach is to select intracellular antigenic targets that are exposed on the cell surface as part of the normal process of protein catabolism and presentation on MHC molecules. Intracellular proteins are usually degraded by the proteasome or endo/lysosomes, and the resulting specific peptide fragments bind to MHC class molecules. These peptide-MHC complexes are displayed at the cell surface where they provide targets for T cell recognition via peptide-MHC T cell receptor (TCR) interaction. The idea of using TCR-like mAb for studying immunobiology and ultimately treating cancer dates back more than a decade and has been nicely reviewed by Dohan and Reiter (Expert Rev Mol Med. 14:e6, 2012.).

Even with the enlarging preclinical demonstration of such TCR-like therapeutic mAb, there remains considerable skepticism as to their promise. The first concern is the low target density on diseased cell surface. With tens of thousands of peptides processed for binding to MHC class molecules within the cell, the likelihood that any one peptide will be expressed on the cell surface in context of HLA molecules in large quantities is small. Many predict that fewer than 10 copies of an individual peptide MHC complex will be presented. That almost all of FDA-approved antibody drugs require tens of thousands of target molecules per cell makes it improbable for such a low density antigen to work. However, for TCR-based T-cell responses, this number appears adequate for effective killing of target cells, based on work from several laboratories. Then why is this not sufficient for mAb mediating human effectors? We observed clearance of disseminated human leukemias in NSG mice with as few as several hundred epitopes present per cell(Dao et al, Science Transl. Med. 5:176r33, 2013.). Moreover, new technology that brings together mAb specifity with T-cell potency have emerged that may make these prejudices against low density targets obsolete. For example, chimeric antigen receptor engineered T cells (CAR T cells) recognize mAb-specific surface targets yet kill cancer cells like a T cell, resulting in patient responses. In addition, bi-specific mAbs, which closely cross-link the target cell to an effector T cell, have also displayed exceptional potency in humans. Second, we and others have seen that the expression of peptide-MHC epitope is not always a few per cell surface, but can be on the order of 5-10,000, a level that is easily approached by conventional mAb therapy or antibody drug conjugates. For example, mAb directed to CD33 have been approved in the treatment of leukemia. Finally, it is possible to upregulate MHC by pharmacologic means, and this may be an approach if antigen density is truly limiting. Therefore, the risk for low antigen density should not discourage the development therapeutic reagents to these targets.

A second hurdle was the notion that peptide-MHC complex internalize poorly or slowly, rendering the use of antibody drug conjugates or radio-conjugates problematic. While this may be true, TCR-like mAb immunotoxins have been shown to be effective in mouse cancer models. In addition, radio-conjugates do not need to be internalized and the potency of alpha-particle emitting and alpha- particle isotope generators, which can kill a cell with a single hit, are certainly potent enough for these epitope densities.

A third criticism is the risk of cross-reactivity with human MHC expressed on all the nucleated cells. This would be a pharmacologic hurdle and possibly a risk for toxicity. Overcoming this problem can be achieved with increasing ease due to the use of phage-display technology and high stringencies in screening to select specific mAb.

A final criticism of the TCR-like mAb approach is that the mAb is HLA-restricted, thereby limiting its use. This is a soft argument in the increasing context of “personalized medicine”, in which each drug will be directed to a subset of patients with a type of cancer. Importantly, given the dominance of just a few HLA worldwide, a TCR-like antibody to an important, widely expressed intracellular oncogenic protein, that crosses many tumor types, could easily still be used in many thousands of patients around the world.

In conclusion, while there are still unanswered questions about the best use, most appropriate targets, and different formats of TCR-like mAb, the lure of finally killing a cell based on the tumor-specific recognition of one of the myriad of important intracellular proteins is strong. In addition, even currently drug-able targets might be suitable mAb targets, as small molecule inhibitors allow escape of the cell via compensatory pathways, relief of feedback inhibition, or mutation of the binding pocket. In contrast, a cytotoxic mAb recognizes *the expression of the epitope* and not its function, making escape unlikely via these mechanisms.

The first human trials of such mAb drugs are likely within the next one to two years. Based on the great success of mAb therapy to date, the selectivity and lack of toxicity, versatility, and ease of development, we are confident that that the reach of the arms of mAb will get longer.

